# Validation of sTREM-1 and IL-6 based algorithms for outcome prediction of COVID-19

**DOI:** 10.1186/s12879-023-08630-0

**Published:** 2023-09-26

**Authors:** Mathias Van Singer, Thomas Brahier, Jana Koch, Pr. Olivier Hugli, Andrea M. Weckman, Kathleen Zhong, Taylor J. Kain, Aleksandra Leligdowicz, Enos Bernasconi, Alessandro Ceschi, Sara Parolari, Danielle Vuichard-Gysin, Kevin C. Kain, Werner C. Albrich, Noémie Boillat-Blanco

**Affiliations:** 1 Infectious Diseases Service, University Hospital of Lausanne, Lausanne, Switzerland; 2 Division of Infectious Diseases and Hospital Epidemiology, Cantonal Hospital St.Gallen, St.Gallen, Switzerland; 3 Emergency Department, University Hospital of Lausanne, Lausanne, Switzerland; 4 Tropical Disease Unit, Department of Medicine, Sandra Rotman Centre for Global Health, University of Toronto, University Health Network-Toronto General, Toronto, ON Canada; 5 Department of Medicine, Western University, London, Ontario, Canada; 6 Division of infectious diseases, Ente Ospedaliero Cantonale, University of Geneva and University of Southern Switzerland, Lugano, Lugano, Switzerland; 7 Ente Ospedaliero Cantonale (EOC), University Hospital Zurich and University of Southern Switzerland, Lugano, Switzerland; 8 Department of Infectious Diseases and Hospital Epidemiology, Cantonal Hospital Muensterlingen, Thurgau Hospital Group, Muensterlingen, Switzerland

**Keywords:** Biomarkers, Validation study, COVID-19, sTREM-1, Clinical support decision tool

## Abstract

**Background:**

A prospective observational cohort study of COVID-19 patients in a single Emergency Department (ED) showed that sTREM-1- and IL-6-based algorithms were highly predictive of adverse outcome (Van Singer et al. J Allergy Clin Immunol 2021). We aim to validate the performance of these algorithms at ED presentation.

**Methods:**

This multicentric prospective observational study of PCR-confirmed COVID-19 adult patients was conducted in the ED of three Swiss hospitals. Data of the three centers were retrospectively completed and merged. We determined the predictive accuracy of the sTREM-1-based algorithm for 30-day intubation/mortality. We also determined the performance of the IL-6-based algorithm using data from one center for 30-day oxygen requirement.

**Results:**

373 patients were included in the validation cohort, 139 (37%) in Lausanne, 93 (25%) in St.Gallen and 141 (38%) in EOC. Overall, 18% (93/373) patients died or were intubated by day 30. In Lausanne, 66% (92/139) patients required oxygen by day 30. The predictive accuracy of sTREM-1 and IL-6 were similar compared to the derivation cohort. The sTREM-1-based algorithm confirmed excellent sensitivity (90% versus 100% in the derivation cohort) and negative predictive value (94% versus 100%) for 30-day intubation/mortality. The IL-6-based algorithm performance was acceptable with a sensitivity of 85% versus 98% in the derivation cohort and a negative predictive value of 60% versus 92%.

**Conclusion:**

The sTREM-1 algorithm demonstrated good reproducibility. A prospective randomized controlled trial, comparing outcomes with and without the algorithm, is necessary to assess its safety and impact on hospital and ICU admission rates. The IL-6 algorithm showed acceptable validity in a single center and need additional validation before widespread implementation.

## Background

The coronavirus disease 2019 (COVID-19) pandemic has challenged health care systems over the world since its appearance in Wuhan in December 2019 [1]. The development of prognostic tools to help clinicians recognize patients at risk of unfavorable outcome at presentation is essential to allocate medical resources to appropriate patients [2].

Many host biomarkers have been associated with COVID-19 disease severity including soluble triggering receptor expressed on myeloid cells 1 (sTREM-1) and interleukin-6 (IL-6) [3–6]. Activation of the sTREM-1 signaling pathway on monocytes/macrophages might contribute to the development of a cytokine storm in the context of COVID-19, and justify using sTREM-1 concentrations to predict hospitalization for oxygen therapy, intubation or death [5]. IL-6 is a pro-inflammatory cytokine increased in patients with severe COVID-19 disease that has been used as a prognostic marker and a therapeutic target for blockade of its signaling pathways [7].

Our team showed in a prospective observational cohort study of COVID-19 patients recruited in the Emergency Department (ED) of Lausanne University Hospital that sTREM-1- and IL-6-based algorithms were highly predictive of adverse outcome [5]. Confirmation of the algorithms predictive value is necessary before their implementation as triage tools [5].

Since post-acute sequelae of COVID-19 (PASC) are reported in ~ 10% of patients with COVID-19 infection, their early detection by initial biomarker levels would be of great additional interest [8].

In the current study, we aimed to externally validate the performance of the sTREM-1 based algorithm at ED presentation, for 30-day intubation/mortality as well as the IL-6 based algorithm for 30-day oxygen requirement. We also aimed to evaluate the predictive value of sTREM-1 and IL-6 for PASC.

## Methods

### Study design and participants

This multicenter prospective observational study of PCR-confirmed COVID-19 adult patients was conducted in the ED of three Swiss hospitals (Lausanne University Hospital, Kantonsspital St.Gallen, a network of four regional hospitals in Southern Switzerland part of the Ente Ospedaliero Cantonale (EOC)). Patients’ data from the three centers were retrospectively completed and merged to form a multicenter cohort used to validate the previously described algorithms, which were derived from a cohort of patients recruited in the ED of the Lausanne University Hospital during the first wave of the pandemic, between February 6 and April 3, 2020 [5].

The validation multicenter cohort was constituted of three cohorts: patients included during the second and third wave of the pandemics between August 18, 2020, and June 10, 2021 in the ED of the Lausanne University Hospital, patients included in the EDs of EOC between March 10 and April 17, 2020 (first wave) and of Kantonsspital St.Gallen between June 7 and November 11, 2020 (second wave). Inclusion methodology was identical to the one used for the derivation cohort [5].

Patients’ demographics, comorbidities, symptoms, vital signs were recorded.

### Host biomarkers

Plasma samples were prospectively collected in the ED of the Lausanne University Hospital, as previously described [5], and serum samples were collected in EOC and St.Gallen hospitals EDs within 24 h after admission. All samples were stored at -80° and retrospectively analyzed head-to-head on a Luminex platform to measure IL-6 and sTREM-1 (R&D Systems, Minneapolis, MN; custom plate, 1:2 dilution). As the samples of the derivation cohort were previously analyzed on an Ella platform, a correction factor was applied on the IL-6 results of the validation cohort, as previously described [9]. In the absence of a validated correction factor between Luminex and ELLA platforms for sTREM-1, this biomarker was measured in the samples of the derivation cohort using the Luminex platform in order to limit potential measurement biases.

### Post-acute sequelae of COVID-19

To assess the post-acute sequelae of COVID-19 (PASC), a comprehensive survey was developed and sent once by mail to patients that presented to the Emergency Department between June and November 2020 at St. Gallen Kantonsspital with PCR-confirmed COVID-19 and between February 2020 and February 2021 at the Lausanne University Hospital Emergency Department (N = 1598). Of those, 474 patients submitted evaluable feed-back. All patients who responded to the survey and whom data on IL-6 and sTREM-1 plasma/serum concentrations were available were included for analysis. Questions were asked 13 to 18 months after confirmed SARS-CoV-2 infection for patients from St Gallen and 12 months after infection for patients from Lausanne.

Long-term outcomes were categorized in no PASC vs. those with PASC (if any PASC category present) with the latter divided into three main domains (neurological and cardiopulmonary) to capture the prominent long-term effects of COVID-19.

To determine the frequency of neurological effects, we listed 25 symptoms and asked whether these symptoms had occurred. Each symptom had to be rated twice, at the time of the infection and at the time of the survey. To assess the cardiopulmonary status, we applied the New York Heart Association (NYHA) classification as well as the Canadian Cardiovascular Society (CCS) classification (22 items overall) to determine the presence and extent of cardiopulmonary complaints twice, within 6 months prior to infection and at the time of the survey.

### Definition of PASC

In order to determine the presence of PASC we categorized the symptoms into two different phenotypes: (1) *Neurological* PASC defined by the presence of anosmia, dysgeusia, “pressure” or “fog” in the head, the feeling of being slowed down, concentration problems or forgetfulness, as these seven characteristics were the most frequently occurring among all patients in our cohort. And (2) *Cardiopulmonary* PASC defined as an increase in NYHA class and/or CCS by ≥ 1 point.

### Statistical analyses

We used the same primary outcome as described in the algorithm derivation study consisting of 30-day intubation/mortality. For the Lausanne validation cohort (patients included during the second and third waves of the pandemics) only, a secondary outcome was evaluated: 30-day oxygen requirement (all patients hospitalized with oxygen requirement) [5]. This item was not available in the two other cohorts.

Patients with neurological or cardiopulmonary symptoms (see definitions above) at 12–18 months were classified as having PASC.

Differences between groups were evaluated by 1-way ANOVA, Kruskal-Wallis, or chi-square tests, as appropriate. A two-sided P value < 0.05 was considered indicative of statistical significance.

We first updated the classification and regression tree analysis (CRT) of the derivation cohort, as previously described [5], to determine the adapted cut-off for the sTREM-1 based algorithm using the measurements on the Luminex platform. The updated sTREM-1 cut-off was at 225 pg/mL (compared with 689 pg/ml when measured with the ELLA platform). The respiratory rate cut-off remained unchanged as determined by CRT analysis in the previous study [5]. The prognostic performance of the updated algorithm for 30-day intubation/mortality was similar to the previous CRT model [5].

In a second step, we assessed the diagnostic accuracy of sTREM-1 and IL-6 in the multicenter validation cohort by calculating the area under the receiver-operating characteristic curve (AUROC) for the 30-day intubation/mortality and for the PASC outcome, as previously described [5].

In a third step, we determined the predictive accuracy of the sTREM-1 based algorithm (including first respiratory rate with a cut-off point at 24/min and, second, sTREM-1 with a cut-off at 225 pg/mL), for the multicenter validation. We also determined the performance of the IL-6 based algorithm (including IL-6 with a cut-off at 15.1 pg/mL), using the Lausanne validation cohort solely, as data on oxygen requirement were unavailable in the St. Gallen and EOC cohorts.

For exploratory purposes, we performed another classification and regression tree analysis (CRT), as described [5], including all vital signs, clinical severity scores, and biomarkers, to determine if another algorithm could be determined with the data from all the centers to predict 30-day intubation/mortality.

All analyses were performed with R Core Team (2019), IBM SPSS version 26 and 29 (IBM Corporation, Armonk, NY) and Excel for Windows.

### Consent for publication

All included patients signed an informed consent form.

### Ethics approval

The Ethics Committees of the canton of Vaud (CER-VD 2019–02283) and of East Switzerland (BASEC 2020–03059 EKOS 20/244) gave ethical approval.

## Results

### Demographics, clinical characteristics and outcome comparison between cohorts

373 patients were included in the multicenter validation cohort, 139 (37%) in Lausanne, 141 (38%) in Ente Ospedaliero Cantonale (EOC) and 93 (25%) in St. Gallen. Overall, 18% (68/373) patients died or were intubated by day 30: 16% (22/139) in Lausanne, 13% (19/141) in EOC and 29% (27/93) in St. Gallen. In Lausanne, 66% (92/139) patients required oxygen by day 30.

Among included patients, long-term follow-up data for PASC were available for 72 patients in St. Gallen and 45 in Lausanne. PASC was present in 62% of patients, with neurological and cardiopulmonary predominance in 47 (76%) and 11 (18%), respectively.

Table 1 and 2 presents patients’ demographics, clinical characteristics and outcome in the derivation cohort, the validation cohort and in the different centers of the multicenter validation cohort. Patients’ characteristics differed between centers of the validation cohort regarding age and the proportion of patients with comorbidities. The proportion of patients meeting the primary outcome did not differ between the derivation and validation cohorts (p = 0.43) although there was a difference between centers (16% in Lausanne vs. 13% in EOC vs. 29% in St. Gallen; p = 0.007).

**Table 1:** Characteristics of study participants at inclusion in the ED for patients of the derivation and the validation cohorts

	Derivation cohort N = 76	Validation cohort N = 373			
	Lausanne (February-April 2020) ( n = 76)	Lausanne (August 2020-June 2021) (n = 139)	EOC (n = 141)	St. Gallen (n = 93)	p value between centers of the validation cohort
Female, n (%)	43 (57)	50 (36)	55 (39)	20 (21)	0.016
Age, mean (SD)	62 (17)	63 (16)	67 (13)	67 (13)	0.029
Cardiovascular disease ^a^, n (%)	10 (13)	18 (13)	36 (25)	36 (42)	< 0.001
Diabetes, n (%)	17 (22)	34 (24)	27 (19)	35 (41)	0.001
Active cancer, n (%)	3 (4)	4 (3)	17 (12)	11 (13)	0.008
Temperature > 38 °C, n (%)	62 (83)	35 (25)	114 (81)	No data	
Cough, n (%)	68 (91)	119 (86)	95 (67)	No data	
Dyspnea, n (%)	58 (76)	124 (89)	76 (54)	No data	
Respiratory rate (vpm), median (IQR)	23 [18, 28]	24 [20, 29]	24 [22, 28]	24 [19, 30]	0.309
30-d intubation, n (%)	10 (13)	10 (7)	19 (13)	15 (16)	0.087
30-d death, n (%)	12 (16)	12 (9)	0 (0)	20 (21)	< 0.001
30-d intubation/death, n (%)	17 (22)	22 (16)	19 (13)	27 (29)	0.007

^a^ Heart failure, coronary disease

Missing values: temperature > 38°C 94, cough 93, dyspnea 93, respiratory rate 32, Active cancer 9, cardiovascular disease 8, diabetes 8

*Differences between the 3 groups evaluated by 1-way ANOVA, Kruskal-Wallis, or x2, as appropriate

**Table 2:** Characteristics of study participants at inclusion in the ED for patients of the derivation and the validation cohorts

	All N = 449	Derivation cohort N = 76	Validation cohort N = 373			
Female, n (%)	168 (37)	43 (57)	125 (33)	0.129		
Age, mean (SD)	65 (15)	62 (17)	66 (14)	0.053		
Cardiovascular disease ^a^, n (%)	100 (23)	10 (13)	90 (25)	0.043		
Diabetes, n (%)	113 (25)	17 (22)	96 (26)	0.569		
Active cancer, n (%)	35 (8)	3 (3.9)	32 (8.8)	0.235		
Temperature > 38 °C, n (%)	211 (60)	62 (83)	149 (53)	< 0.001		
Cough, n (%)	282 (80)	68 (91)	214 (76)	0.011		
Dyspnea, n (%)	258 (72)	58 (76)	200 (71)	0.483		
Respiratory rate (vpm), median (IQR)	24 [20, 30]	23 [18, 28]	24 [20, 30]	0.015		
30-d intubation, n (%)	54 (12)	10 (13)	44 (12)	0.889		
30-d death, n (%)	44 (9.8)	12 (16)	32 (8.6)	0.072		
30-d intubation/death, n (%)	85 (19)	17 (23)	68 (18)	0.431		

^a^ Heart failure, coronary disease

Missing values: temperature > 38°C 94, cough 93, dyspnea 93, respiratory rate 32, Active cancer 9, cardiovascular disease 8, diabetes 8

*Differences between the 3 groups evaluated by 1-way ANOVA, Kruskal-Wallis, or x2, as appropriate

Patients with adverse outcome (30-day intubation/mortality) were older (p < 0.001), had more often cardiovascular disease (p = 0.049) and a higher respiratory rate (p < 0.001).

### Il-6 and sTREM-1 predict 30-day intubation/mortality in the validation multicenter cohort

**Fig. 1:**
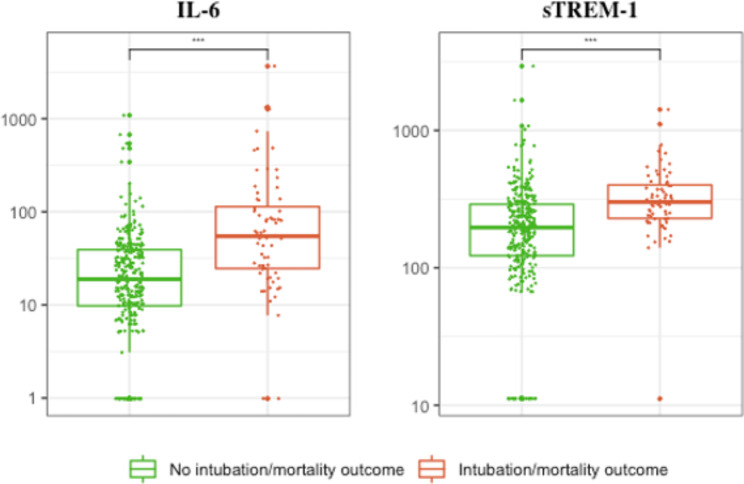
Concentration of IL-6 and sTREM-1 at inclusion in the emergency department according to 30-day intubation/mortality in the multicenter validation cohort (N = 373)

**Fig. 2:**
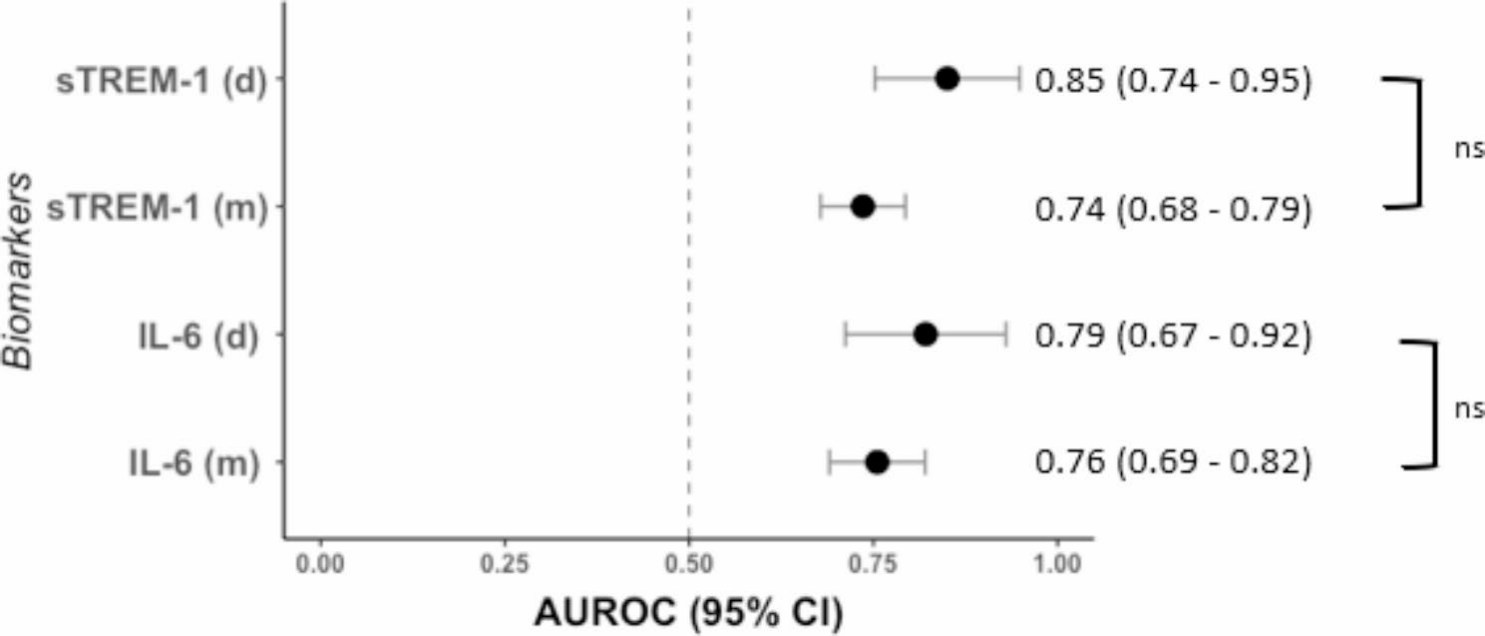
Prognostic accuracy of IL-6 and sTREM-1 measured at the ED for 30-day intubation/mortality in the derivation cohort (d) and in the multicenter validation cohort (m). Nonparametric ROC curves were generated and compared between the derivation and multicenter validation cohort using the DeLong method. Area under the receiver-operating characteristic curves (AUROC) with 95% confidence intervals (CI) are presented on the right of the forest plot

Overall, IL-6 and sTREM-1 levels were significantly higher in patients meeting the primary outcome of intubation/mortality in the multicenter validation cohort (Fig. 1). The predictive accuracy of sTREM-1 and IL-6 were similar in the multicenter validation cohort compared with the derivation cohort (Fig. 2).

### Multicenter validation of sTREM-1 based algorithm

We tested the sTREM1 based algorithm in the multicenter validation cohort for prediction of 30-day intubation/mortality, after excluding 32 patients with missing respiratory rate. We obtained a sensitivity of 90%, specificity of 35%, positive likelihood ratio (PLR) of 1.37 and negative likelihood ratio (NLR) of 0.30 confirming generalizability and good performances, particularly with a high sensitivity and moderate NLR, for a safe management of COVID-19 patients. These results were similar to the derivation cohort (updated values from the original derivation study after biomarkers dosing on the Luminex platform and new classification and regression tree analysis (CRT) analysis): sensitivity: 100%, specificity: 62%, PLR: 2.66 and NLR: 0.0) demonstrating good reproducibility.

### Validation of the IL-6-based algorithm to predict 30-day oxygen requirement

We tested the IL-6 based algorithm to predict 30-day oxygen requirement solely on the Lausanne validation cohort and had a sensitivity of 85%, specificity of 45%, PLR of 1.53 and NLR of 0.34. This performance was lower than in the derivation cohort (sensitivity 98%, specificity 50%, PLR 1.96 and NLR 0.04) but demonstrated acceptable reproducibility. Indeed, an 85% sensitivity in predicting low-severity events like oxygen requirement is considered acceptable.

### Classification and regression tree analysis in the multicenter validation cohort

**Fig. 3:**
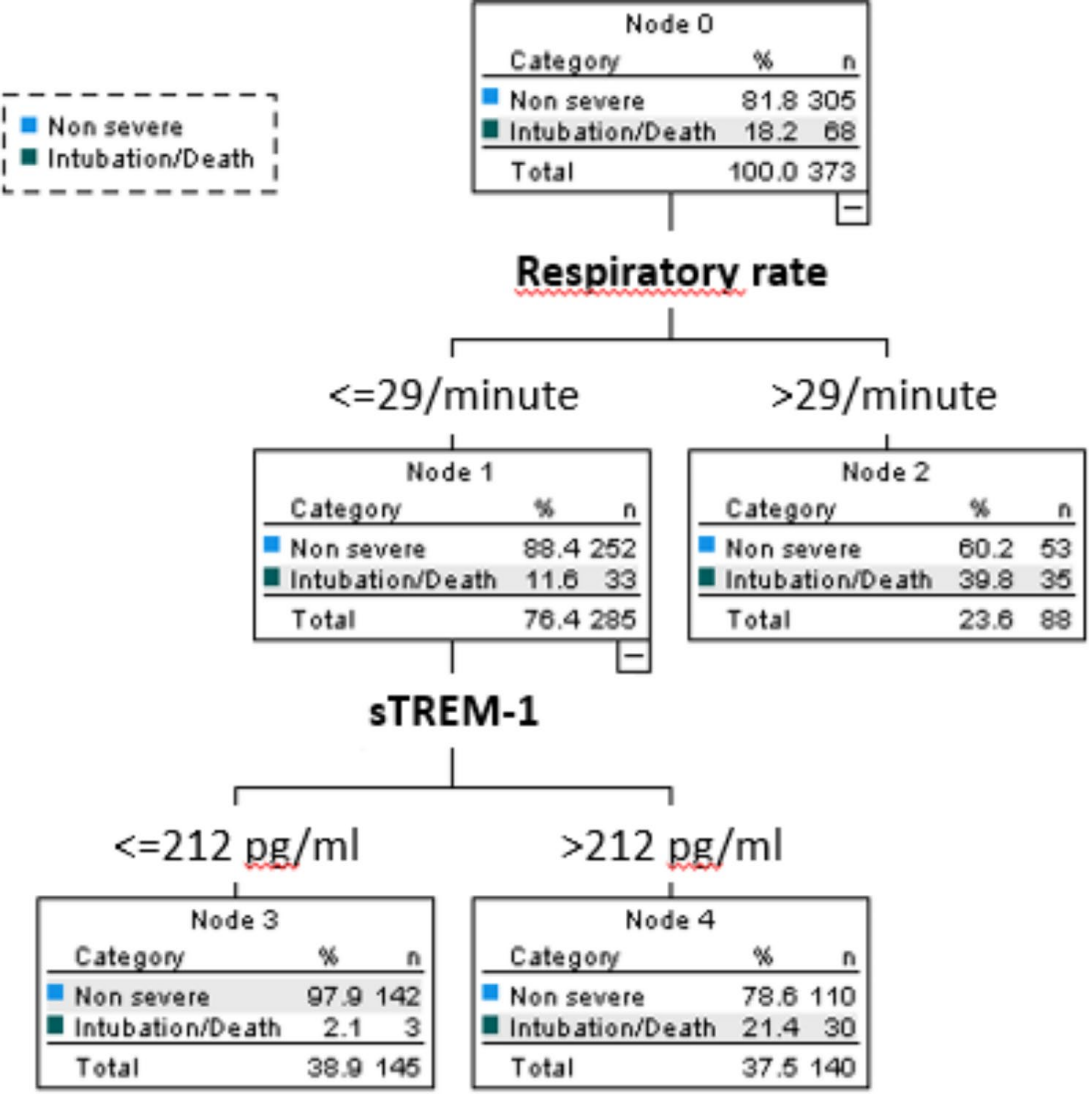
Classification and regression tree analysis (CRT) algorithm to predict 30-day intubation/mortality in patients of the multicenter validation cohort. CRT analysis including all biomarkers and vital signs identified the model including a combination of respiratory rate and sTREM-1. The cost of misclassifying a patient who was intubated or died was designated as 10 times the cost of misclassifying a patient who survived without intubation. Cut-off points selected by the CRT analysis are shown between the parent and child nodes. The outcome prediction of the model is indicated below each terminal node

We performed a new CRT analysis in the multicenter validation cohort (Fig. 3), which resulted in a very similar visual decision-making tree compared with the one of the derivation cohort. It included first respiratory rate and then sTREM-1. Its prognostic performance was similar to the previous CRT model (sensitivity 96%, specificity 47%, PLR 1.81 and NLR 0.09). Similar to the previous decision tree (from the derivation cohort), sTREM-1 enabled the detection of patients with a lower respiratory rate (between the identified cut-off) who were at an elevated risk of intubation or mortality.

### Lack of PASC prediction

Neither IL-6 (area under the receiver-operating characteristic curve **(**AUROC) 0.562; 95%CI 0.402–0.721) nor sTREM-1 (AUROC 0.499; 95%CI 0.33–0.668) was able to accurately identify patients with PASC (evaluated 12 to 18 months after confirmed SARS-CoV-2).

## Discussion

In this multicenter prospective cohort study of SARS-CoV-2 infected patients, we validated our algorithm based on the respiratory rate and sTREM-1 using newly acquired independent data to predict 30-day intubation and mortality. We also validated the IL-6 algorithm to predict 30-day oxygen requirement using single center data (Lausanne) [5]. However, neither of the tested biomarkers was predictive of development of PASC.

Triage tools to help ED clinicians identify patients at risk of adverse outcome are essential to optimize resource allocation, especially in the context of a global pandemic [10]. Increased sTREM-1 levels are associated with poor clinical outcome in patients with COVID-19, as shown in various studies and a meta-analysis. It is therefore a good candidate as triage tool [4–6, 11–13]. IL-6 plays a role in the genesis of the pro-inflammatory lung-systemic loop leading to a cytokine storm syndrome and acute respiratory distress syndrome [14]. It has been associated with adverse outcome in patients with COVID-19 and might be used to guide clinicians in the identification of patients with severe COVID-19 early in their disease course [15, 16].

Our study had several limitations. First, a different technique for the measurement of biomarkers concentrations with the derivation cohort that required a repeated sTREM-1 measurement with the Luminex platform and an updated sTREM-1-based algorithm based on a new cut-off. Second, plasma samples were used in the derivation and Lausanne validation cohorts, while serum samples were used in EOC and St. Gallen. This could have affected the results of our study. Third, recruitment processes differed between study sites. For example, in EOC and St. Gallen, only admitted patients were included, while in Lausanne, outpatients were also eligible, which could explain the differences of characteristics and main outcome frequency seen in Table 1.1. Finally, the lack of PASC prediction by biomarkers might be related to our small sample size, although a true lack of association is a possibility.

## Conclusion

The respiratory rate and sTREM-1 based algorithm demonstrated good reproducibility and generalizability in our multicenter validation study. This provides additional support for its potential real-life use in the ED for early triage of COVID-19 patients and making decisions regarding hospital admission and/or the need for intensive monitoring.

The IL-6 based algorithm for 30-day oxygen requirement prediction showed acceptable local validity and needs to be validated in external centers prior to widespread implementation in the ED.

These biomarker-based algorithms could provide guidance as decision-making tools in the ED. However prospective randomized controlled trial (with / without the algorithms) must be done to evaluate its safety and impact on the rate of hospital and ICU admission in actual practice.

## Data Availability

The datasets analyzed during the current study are available from the corresponding author on reasonable request.

## Abbreviations

sTREM-1: soluble triggering receptor expressed on myeloid cells 1
IL: interleukin
COVID-19: Coronavirus disease 2019
ED: Emergency Department
PASC: post-acute sequelae of COVID-19
EOC: Ente Ospedaliero Cantonale
PLR: positive likelihood ratio
NLR: negative likelihood ratio
CRT: classification and regression tree analysis
AUROC: area under the receiver-operating characteristic curve
